# Arthroscopy or ultrasound in undergraduate anatomy education: a randomized cross-over controlled trial

**DOI:** 10.1186/1472-6920-12-85

**Published:** 2012-09-09

**Authors:** Matthias Knobe, John Bennet Carow, Miriam Ruesseler, Benjamin Moritz Leu, Melanie Simon, Stefan K Beckers, Alireza Ghassemi, Tolga T Sönmez, Hans-Christoph Pape

**Affiliations:** 1 Department of Orthopaedic Trauma, Medical Faculty, RWTH Aachen University, 30 Pauwelsstreet, Aachen 52074, Germany; 2 Department of Trauma Surgery, Medical Faculty, Johann Wolfgang Goethe Hospital, Frankfurt University, 7 Theodor Stern Kai, Frankfurt am Main 60590, Germany; 3 Dean' office for study affairs, Medical Faculty, RWTH Aachen University, 30 Pauwelsstreet, Aachen 52074, Germany; 4 AIXTRA – Aix-la-Chapelle Centre for Interdisciplinary Training in Medical Education, RWTH Aachen University, 30 Pauwelsstreet, Aachen 52074, Germany; 5 Department of Oral and Maxillofacial and Plastic Facial Surgery, Medical Faculty, RWTH Aachen University, 30 Pauwelsstreet, Aachen 52074, Germany

**Keywords:** Arthroscopy, Education, Anatomic competence, Randomized controlled trial, Knee joint, Shoulder joint, Students, Medical, Musculoskeletal ultrasound

## Abstract

**Background:**

The exponential growth of image-based diagnostic and minimally invasive interventions requires a detailed three-dimensional anatomical knowledge and increases the demand towards the undergraduate anatomical curriculum. This randomized controlled trial investigates whether musculoskeletal ultrasound (MSUS) or arthroscopic methods can increase the anatomical knowledge uptake.

**Methods:**

Second-year medical students were randomly allocated to three groups. In addition to the compulsory dissection course, the ultrasound group (MSUS) was taught by eight, didactically and professionally trained, experienced student-teachers and the arthroscopy group (ASK) was taught by eight experienced physicians. The control group (CON) acquired the anatomical knowledge only via the dissection course. Exposure (MSUS and ASK) took place in two separate lessons (75 minutes each, shoulder and knee joint) and introduced standard scan planes using a 10-MHz ultrasound system as well as arthroscopy tutorials at a simulator combined with video tutorials. The theoretical anatomic learning outcomes were tested using a multiple-choice questionnaire (MCQ), and after cross-over an objective structured clinical examination (OSCE). Differences in student’s perceptions were evaluated using Likert scale-based items.

**Results:**

The ASK-group (n = 70, age 23.4 (20–36) yrs.) performed moderately better in the anatomical MC exam in comparison to the MSUS-group (n = 84, age 24.2 (20–53) yrs.) and the CON-group (n = 88, 22.8 (20–33) yrs.; p = 0.019). After an additional arthroscopy teaching 1% of students failed the MC exam, in contrast to 10% in the MSUS- or CON-group, respectively. The benefit of the ASK module was limited to the shoulder area (p < 0.001). The final examination (OSCE) showed no significant differences between any of the groups with good overall performances. In the evaluation, the students certified the arthroscopic tutorial a greater advantage concerning anatomical skills with higher spatial imagination in comparison to the ultrasound tutorial (p = 0.002; p < 0.001).

**Conclusions:**

The additional implementation of arthroscopy tutorials to the dissection course during the undergraduate anatomy training is profitable and attractive to students with respect to complex joint anatomy. Simultaneous teaching of basic-skills in musculoskeletal ultrasound should be performed by medical experts, but seems to be inferior to the arthroscopic 2D-3D-transformation, and is regarded by students as more difficult to learn. Although arthroscopy and ultrasound teaching do not have a major effect on learning joint anatomy, they have the potency to raise the interest in surgery.

## Background

The exponential growth of new techniques of image presentation and minimally invasive surgical techniques increasingly requires a three-dimensional anatomical knowledge. A profound and in-depth training in anatomy during undergraduate medical studies has therefore gained importance [1]. Results of previous studies indicated that students benefit from incorporating virtual multidimensionality in anatomy training [2-5]. Trained ultrasound skills do improve the anatomical knowledge uptake among students [6]. The alternating use of ultrasound and anatomy training can improve both entities. Herewith, students receive a profound background that forms the basis for the acquisition of further skills in different subspecialties [7]. Previous studies suggested that students may learn the basics of ultrasound with comparatively little didactic investment [8-11]. However, only two studies specifically evaluated musculoskeletal ultrasound (MSUS) [10,11]. Prior to completing the anatomical training regarding the musculoskeletal system, one should use experienced medical experts or didactically and professionally trained student-teachers in MSUS training [11].

Arthroscopy can increase the learning success in anatomy through its required cognitive 2D-3D-transformation as opposed to simply swotting up on anatomy. The two dimensional view of the arthroscopy requires, among bimanual-visual coordination, the cognitive deepness estimation of the anatomic structures. The ideal method to acquire this complex skill has not been identified yet. It remains unclear whether every student or even prospective surgeon brings along the psychomotor precondition to learn this coordinative challenging ability [12]. The additional use of arthroscopic methods in the anatomical training has not been proven significantly profitable yet. Albeit it has been shown, that student motivation, regarding anatomical training, can be improved with the use of surgical simulators. However, no measurable superior learning success in comparison to illustrated textbooks has been found [13]. During the simulator training, direct anatomic landmarks could be detected faster and better [14]. Hence, the question arises which kind of cognitive 2D-3D-transformation, ultrasound or arthroscopy, is more beneficial for students’ knowledge acquisition in the compulsory dissection course of the curriculum. We conducted the present randomized cross-over controlled trial in order to answer the question: Do short tutorials of ultrasound or arthroscopy increase the anatomical knowledge uptake in comparison to macroscopic cadaver dissection only?

## Methods

### Study design

The musculoskeletal anatomy training starts with attendance in the dissection course at the beginning of the second year. During 2011, all second year medical students were affiliated to this randomized controlled trial. The affiliation to this trial took place due to changes of the curriculum’s schedule and contents. Ethical approval (EK 178/09) was obtained from the Ethics Committee, Medical Faculty, RWTH Aachen University (Chairman G. Schmalzing). The adjustments to the curriculum were developed in close cooperation with the Medical Faculty study Dean’s office and consisted mainly of additional tutorials of ultrasound of the knee (75 min) and shoulder joint (75 min) and tutorials for the introduction of arthroscopy using a simulator (150 min in total each). Simultaneously, the content outline was revised and the lectures on orthopaedic and trauma surgery were optimized and revised accordingly. Through a close cooperation of the project partners it was guaranteed, that all involved departments (trauma surgery and orthopaedics, anatomy) dealt with the topics “shoulder” and “knee” in the first week. All students denied any previous experience with MSUS and arthroscopy in a questionnaire given prior to the beginning of the study.

**Figure 1:**
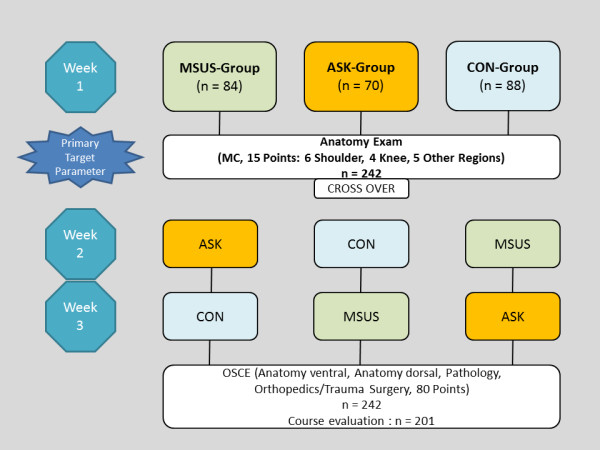
**The flow of participants through each stage of the trial is shown.** ASK: arthroscopy, MSUS: musculoskeletal ultrasound, CON: control-group without additional intervention (only cadaver dissection course), OSCE: objective structured clinical examination.

Participants were randomly assigned following simple randomization procedures (computerized random numbers) to one of the three trial groups (Figure 1). The CON-group served as control group and received their anatomical knowledge solely through the established dissection course. The MSUS-group received additional ultrasound training in the first week of the dissection course, while the ASK-group received additional arthroscopy training using a simulator. Autonomous self-study was not regulated through the trials’ setting.

The MSUS-group was taught by 8 undergraduate medical students in their fifth year who received a special training. The arthroscopy group (ASK) was taught through clinically experienced senior physicians (all male).

### Lecturers training

During a “teach-the-teacher-course” the eight student-teachers received a sound didactical training (four sessions with a duration of 120 min each), including the basics of modern learning concepts (Sandwich theory of a good seminar, Bloom-taxonomy) [15,16], innovative studying techniques (peer-assisted learning (PAL), team-based learning, e-learning) and assessment strategies (OSCE, MCQ). The professional ultrasound training consisted of three sessions (120 min each). Each session started with a 30 min theoretical introduction to the different transducers, sequences of medical examinations, certain indications, statements and standard scan planes; followed by a 90 min practical training including the standard scan planes of the shoulder and knee joint (European League Against Rheumatism [EULAR]) [17]. The medical experts for the arthroscopy education did not receive any additional training. They were highly involved in regular teaching of medical students, each with at least 5 years of teaching experience.

### Intervention

Each training group for the ultrasound and the arthroscopy part consisted of 16–20 students, with a maximum of five students per ultrasound device or arthroscopy simulator. The additional training consisted of two sessions of 75 min for each trial group, divided into a shoulder and a knee joint part.

For the ultrasound training (MSUS), four devices of the Toshiba Medical Systems GmbH (Nemio XG™, Neuss, Germany) with a 10 MHz-linear transducer were used. Students performed sonography scans on each other as opposed to models. Special emphasis was put on the anatomical understanding in two-dimensional (2D) scan planes and the three-dimensional (3D) imagination of the structures.

Arthroscopy training (ASK) was performed on two shoulder and knee models (Arthrex Medical Instruments GmbH, Karlsfeld, Germany). A teaching video was presented to the students, which included a diagnostic circuit (three minutes) through the physiologic joint followed by an illustrated presentation on the most important anatomic landmarks. The video was then repeated once more. In the practical part, students learned the basics of arthroscopy (TelePack X, Karl Storz GmbH & Co. KG, Tuttlingen, Germany) focusing on independent instrument handling without direct view.

### Evaluation

The acquired anatomical knowledge was evaluated in a theoretical exam (multiple choice, MCQ, max. 15 points) after one week. A multiple choice format with a single correct response option and four distractors (“1 of 5”) was used. The MC exam (primary outcome of the trial) included six questions on the topic shoulder, four questions on the knee joint, as well as five questions on anatomic aspects of other body regions (abdomen, hip joint, ankle joint, ischiocrural and fundament muscles). Fifty percent of the questions concerning the shoulder and knee joint were connected to anatomical drawings. A score of at least 60% (9 points) was required to pass the MC exam according to the standing orders of the Medical Faculty. After the exam, students switched groups twice in order to give every student the possibility to attend all additional training (Figure 1). At the end of the dissection course a practical exam (objective structured clinical examination, OSCE) was performed. During the OSCE, anatomy (maximum of 40 points) and pathology (maximum of 20 points) skills were tested during a cadaver dissection. This was followed by a clinical “trauma surgery/orthopaedics” (maximum of 20 points) part.

The students evaluated the course through a standardized questionnaire using a 5-point-Likert-Scale (1 = strongly agree, 2 = agree, 3 = neither agree nor disagree, 4 = disagree, 5 = strongly disagree). Crucial points were the lecturer´s expertise, fun factor, subjective appraisal of the achieved anatomical knowledge, as well as evaluation of the additional multidimensional training through ultrasound and arthroscopy. Furthermore, the subjectively achieved spatial imagination in general and the peer-assisted learning (PAL) concept as part of the ultrasound were evaluated.

### Statistics

After testing for normality (Kolmogorov-Smirnov), differences among students with respect to objective and subjective quantitative parameters (MCQ, OSCE) were calculated using ANOVA. Paired t-tests were used to assess differences, regarding the evaluation of arthroscopy in comparison to ultrasound. Categorical comparisons regarding the MC exam were made using the Chi-squared test (2 groups) and the Fisher´s exact test (3 groups). All tests were two-tailed and assessed at the 5% significance level. Analysis was done with the statistic software SPSS™ 19.0 (SPSS Inc., Chicago, IL, USA).

## Results

### Demographic results

There were 164 female and 78 male students (mean age 23.5 (20–53) years). Eighty-four students were allocated to the MSUS-group (56 female, 28 male, age 24.2 (20–53)), 79 students to the ASK-group (50 female, 20 male, age 23.4 (20–36) years) and 88 students to the CON-group (58 female, 30 male, age 22.8 (20–33) years). There was no difference between groups with respect to demographic parameters.

### Main study parameter

Evaluation of the anatomical knowledge (MCQ) after the first week showed a higher overall knowledge of the ASK-group compared to the MSUS- and CON-group (Table 1; p = 0.019). In the ASK-group 99% of the students passed the MC exam, with only one student who failed. Moreover, 89% of the students in the CON-group and 91% in the MSUS-group, respectively, passed this part of the exam (ASK *vs.* CON: p = 0.024, ASK *vs.* MSUS: p = 0.033, ASK *vs.* MSUS *vs.* CON: p = 0.074). No differences between the trial groups could be detected for the knee and other anatomical regions, which had not been part of the multidimensional presentation. However, students of the ASK-group showed knowledge advantages (ASK *vs.* MSUS *vs.* CON: 5.4 *vs.* 4.8 *vs.* 5.0 points; p < 0.001; Table 1) with respect to the shoulder region.

**Table 1:** Comparison of the three groups regarding the acquired anatomical knowledge at the end of the one week training (MC exam)

**MCQ after 1 week**	**MSUS n = 84**	**ASK n = 70**	**CON n = 88**	**p**
Anatomy (15 questions, 15 points)	11.1 (1.7)	11.9 (1.7)	11.3 (1.8)	0.019
*MC exam passed (n/%)*	76/91	69/99*	79/89	0.074
*- Anatomy shoulder (6 points)*	*4.8 (1.0)*	*5.4 (0.8)*	*5.0 (1.0)*	*< 0.001*
*- Anatomy knee (4 points)*	*2.7 (0.6)*	*2.7 (0.7)*	*2.6 (0.6)*	*0.317*
*- Anatomy rest (5 points)*	*3.5 (1.1)*	*3.7 (0.9)*	*3.7 (1.0)*	*0.456*

* ASK *vs.* CON: p = 0.024; ASK *vs.* MSUS: p = 0.033 (Chi-squared test).

All scores are quoted as arithmetic average (standard deviation).

For the final exam after three weeks (OSCE) no significant differences between groups were detected, with good overall performance of all students (15.9-18.4 points, max. 20 points; Table 2).

**Table 2:** Comparison of the three groups regarding the OSCE results at the end of the three week training

**OSCE after 3 weeks**	**MSUS n = 84**	**ASK n = 70**	**CON n = 88**	**p**
Anatomy ventral (20 points)	16.3 (4.1)	17.4 (4.0)	17.7 (3.1)	0.051
Anatomy dorsal (20 points)	15.9 (4.1)	16.7 (3.6)	16.8 (3.5)	0.252
Pathology (20 points)	17.5 (3.7)	18.4 (3.3)	18.3 (3.4)	0.202
Trauma surgery/Orthopaedics				
(20 points)	17.3 (2.5)	18.0 (2.9)	18.0 (2.5)	0.190

All scores are quoted as arithmetic average (standard deviation).

A total of 83.1% (201 of 242) of participants evaluated the course (Table 3). At this time all three groups had completed all three parts of the training (Figure 1). The multidimensional augmentation of the classic anatomical education was considered very helpful, with a high short-term, but also prognostic long-term learning effect, considering anatomical knowledge of the musculoskeletal system (Table 3). However, there were remarkable differences in students’ perception regarding the arthroscopy and ultrasound training. In comparison to the ultrasound education, arthroscopy training was rated higher, considering the achievable anatomical knowledge and better spatial sense (p = 0.002; p < 0.001). Specific anatomical structures could subjectively better be identified with arthroscopy (p < 0.001). Although the PAL concept was rated to be a good learning method (LS 1.8), student-teachers were allocated a lesser level of competence than staff lecturers (p < 0.001).

**Table 3:** Evaluation of the course parts arthroscopy and sonography after 3 weeks

***Evaluation (Likert-Scale, LS, 1–5)# after course end***	**Arthroscopy**	**Ultrasound**	**p **
Number of evaluation (n)			**201**
The lecturer was competent	1.3 (0.7)	1.7 (0.9)	**< 0.001**
The lecture was fun	1.5 (0.8)	1.5 (0.7)	**0.816**
I have learned alot	1.9 (1.0)	1.9 (0.9)	**0.552**
Theory and practice were well combined	1.6 (0.9)	1.9 (0.9)	**0.001**
The size of the group was optimal	2.3 (1.2)	2.3 (1.3)	**0.631**
The interaction between the group and the lecturer was good	1.6 (0.8)	1.6 (0.8)	**0.691**
Multidimensional augmentation in anatomical education makes sense	1.7 (0.9)	1.8 (0.9)	**0.315**
Structures were difficult to identify	3.2 (1.2)	2.6 (1.2)	**< 0.001**
Many of my questions stayed unanswered	3.9 (0.9)	3.6 (1.0)	**0.004**
I would need more lectures for deepening	2.1 (1.1)	1.8 (1.1	**< 0.001**
Generally the PAL concept is a good teaching method	1.8 (1.0)	1.8 (1.0)	**0.991**
Only a medical expert can teach these contents	3.2 (1.3)	3.6 (1.1)	**< 0.001**
Generally the contents were too comprehensive	4.0 (0.9)	3.7 (1.0)	**0.003**
I could improve my anatomical knowledge	1.9 (1.0)	2.3 (1.1)	**0.002**
The durability of my anatomical knowledge is raised	1.7 (0.9)	1.7 (0.9)	**0.624**
My spatial imagination was improved	1.6 (0.9)	2.0 (1.1)	**< 0.001**
I was better prepared for the practical exam (OSCE)	2.7 (1.2)	2.8 (1.2)	**0.117**
This lecture should later be introduced in the study	3.6 (1.3)	3.6 (1.3)	**0.202**
ASK and MSUS awaked my interest in surgery	2.4 (1.1)	2.4 (1.0)	**0.899**

# Likert-Scale (LS): 1 complete approval – 5 entire rejection.

PAL: peer-assisted learning.

ASK: arthroscopy.

MSUS: musculoskeletal ultrasound.

Questions of the questionnaire were translated from German.

All scores are quoted as arithmetic average (standard deviation).

According to the participants, it is rather possible for student-teachers to teach ultrasound contents than to teach arthroscopic basic skills (p < 0.001; Table 3). The early introduction to arthroscopy and MSUS was considered beneficial to create an interest in surgery (LS 2.6).

## Discussion

In this open randomized cross-over clinical trial among 2^nd^ year medical students, the addition of short educational units of arthroscopy to the macroscopic dissection course increased the anatomical knowledge gain compared to standard anatomy training. The addition of equivalent educational units of musculoskeletal ultrasound (MSUS) to the standard anatomy training using peer-assisted learning (PAL) did not improve anatomical skills. Despite a statistically significant difference in performance between the study groups regarding the MC exam, the absolute differences were moderate. However, after additional arthroscopy teaching only 1% of students failed the MC exam, in contrast to 10% in the MSUS- or CON-group, respectively.

The benefit of the ASK module was limited to the shoulder area. The multidimensional augmentation of the standard anatomy lecture with arthroscopy and ultrasound was considered beneficial by the students regarding the acquisition of anatomical knowledge of the musculoskeletal system. When asked to compare, students preferred arthroscopy due to a better anatomical orientation and a higher gain in anatomical knowledge as well as in spatial imagination.

The benefit of virtual multidimensionality in anatomical education has been demonstrated before [1]. For this reason our study investigates, which particular kind of cognitive 2D-3D-transformation, ultrasound or arthroscopy, enhances anatomical knowledge during the curricular dissection course. The musculoskeletal ultrasound (MSUS) has already been incorporated successfully into the curricular anatomical education [18]. Teaching these skills at an early level may improve medical expertise in diagnostic methods and may improve the quality of patient care [2]. Ultrasound is a rapidly available and cost-saving instrument, and is a perfect teaching tool for medical students [7]. Contrary to the results of other trials [2-6] students in this study did not benefit from the additional ultrasound courses of the musculoskeletal system with regard to anatomical skills. A possible explanation might be the fact that we used student-teachers as lecturers. Minimally instructed student-teachers can be employed to teach anatomically skilled students, whereas medical experts should be used in earlier stages of anatomical education [11]. Tolsgaard *et al.* and Shiozawa *et al.* postulated that the specific training of student-teachers was an absolute necessity for success in the environment of peer-assisted learning [19,20]. Therefore, we included student-teachers who received an extensive training in education theory (implementation of a teach-the-teacher course) in our study. Possibly, the results of Tolsgaard and Shiozawa cannot be transferred to MSUS. Because of the strong operator-dependence, an adequate training is highly important to guarantee a firm and competent MSUS application [21].

However, we showed in our study, that arthroscopy tutorials using simulators held by medical experts moderately increased the anatomical knowledge gain in comparison to solely macroscopic dissection. Arthroscopy models can be very useful, regarding the anatomical education of students without any previous experiences [14]. To the best of our knowledge, our trial is the first randomized study evaluating the direct anatomical knowledge benefit of arthroscopic skill training in comparison to a control group without any intervention. Until now it has only been shown that students’ motivation towards anatomical education could be increased by the use of surgical simulators [13]. It has been demonstrated that not every student has the psychomotor precondition to learn complex arthroscopy skills [12].

Our results show that a multimedia presentation and consecutive “hands on” training supported the anatomical learning efficiency. Translating a two dimensional view into three-dimensional spatial orientation requires a bimanual psychomotor activation, estimation of depth and coordination of the visual and tactile sense. These complex coordinative skills may act as important memory anchors. Other approaches to virtual three-dimensional visualization have also been able to show potential additional benefit for the anatomic education [22,23].

One recent study on virtual dissection software as opposed to a cadaver-based course reported that students perceived that the virtual approach is highly valuable in their learning of anatomy [24].

Students in our cohort reported that structures were easier identifiable using arthroscopy and that arthroscopy training was associated with a subjectively higher learning effect in comparison to ultrasound. Especially spatial imagination was described as clearly improved.

The impact of the different teaching methods (medical experts *vs.* student-teachers) can not be estimated entirely. Even though peer-assisted learning is regarded as a good teaching concept, medical experts of the arthroscopy module scored higher than the student-teachers of the MSUS module in terms of level of competence. One aspect might be the steep learning curve and the complexity of MSUS [17,21]. According to the students’ opinion, arthroscopy and MSUS have the potency to raise interest in surgery. This is in line with an earlier report by our study group that showed that students develop an interest in orthopaedic trauma at a very early level but somehow lose it over time due to negative experience during their clinical training [25]. In times of shortage of young academics, this is a very important factor that is amenable to intervention.

The question, why students particularly profit from an arthroscopy augmented anatomical education for the shoulder as opposed to the knee joint, seems difficult to answer. On the one hand the level of musculoskeletal education varies according to anatomical regions, on the other hand the anatomical complexity appears to be region depending as well [26]. Day and Yeh showed that students’ confidence in their own examination techniques are clearly reduced at the shoulder while their confidence is above average regarding the knee joint [26]. Since students are apparently very familiar with examination techniques of the knee, an additional arthroscopic intervention may not lead to an additional knowledge gain. In contrast, the anatomy of the shoulder is more difficult to understand and ultrasound examinations of the shoulder have been attributed one of the steepest learning curves [27].

### Limitations

Our study is subject to a number of limitations. First: Despite significance there were only slightly differences between the study groups in terms of the 15-point MCQ and its sub-analysis, and one must be wary of drawing major definitive conclusions from these test results. A more comprehensive knowledge-based shoulder and knee anatomy test may have demonstrated differences more clearly.

This was a single-centre study. Results may differ in different organizational or didactical settings. Furthermore, we did not assess the level of any anatomical knowledge or skills concerning ultrasound and arthroscopy techniques acquired prior to the intervention. However, according to the curriculum, students had not received any anatomical training on the musculoskeletal system prior to the study and students denied any such qualifications in the questionnaire. The anatomical outcome measures only refer to the shoulder and knee joint and an extrapolation of our results to other anatomic regions is not valid.

We could not control for autonomous self-study and students’ motivation which might have influenced the final test results. We do not see this as a threat to internal validity since selection bias was controlled for by including a large number of participants and using methods of complete random sampling. The study guideline allowed students to miss two classes during the entire 3-week dissection course. Frequency and timing of absence had no significant influence on the final result.

Although the course evaluation did not show any differences between teachers, a certain amount of variability in the level of competence of the student-teachers can not be ruled out.

## Conclusions

Incorporating arthroscopy education into anatomy training in undergraduate medical school, showed a moderate knowledge increasing effect, especially in anatomically complex joint sections, such as the shoulder region. In comparison to the standard dissection course, the supplementary teaching of basic skills in the musculoskeletal ultrasound using professionally and didactically trained student-teachers, did not improve knowledge uptake. Even though students prefer arthroscopy, they consider the early introduction to musculoskeletal ultrasound as useful and attractive. Although arthroscopy and ultrasound teaching do not have a major effect on learning joint anatomy, they have the potency to raise the interest in surgery. In times of shortage of young academics, this is very important and consistent with the awareness, that already students in an early education level develop a vast interest in the surgery of the musculoskeletal system.

## Competing interests

The authors declare that they have no competing interests.

## Authors’ contributions

MK had full access to all of the data in the study and takes responsibility for the integrity of the data and the accuracy of the data analysis. All authors meet all three of the requirements for authorship. JBC, MR, SKB, AG and BML were highly involved in the planning and execution of this study. MS organized the project by order of the dean´ office of study affairs. Furthermore JBC, MR, BML, SKB, AG and MS were highly involved in the acquisition of data and in the process of data interpretation. HP and TTS made a significant contribution to the analysis and interpretation of data. Furthermore they took part in the manuscript review process and revised it critically. In this way they provided an important intellectual content in line with study execution. MK acted as the initiator of the study and was highly involved in the advancement of the conception. Together with JBC, MR and BML he was highly involved in the process of data acquisition, in the process of the statistic converting of data and in the process of data interpretation. All authors read and approved the final manuscript.

## Pre-publication history

The pre-publication history for this paper can be accessed here:

http://www.biomedcentral.com/1472-6920/12/85/prepub
